# RATES OF VISION AND HEARING IMPAIRMENT RELATED TO COGNITIVE DIFFICULTIES AMONG OLDER ADULTS IN MISSISSIPPI COUNTIES

**DOI:** 10.1093/geroni/igad104.2953

**Published:** 2023-12-21

**Authors:** Yan-Jhu Su, Taylor Jansen, Chae Man Lee, Nina Silverstein, Elizabeth Dugan, Shu Xu, Kina White

**Affiliations:** University of Massachusetts Boston, Boston, Massachusetts, United States; University of Massachusetts Boston, Boston, Massachusetts, United States; Univ of CT Health, Farmington, Connecticut, United States; University of Massachusetts Boston, Boston, Massachusetts, United States; University of Massachusetts, Boston, Boston, Massachusetts, United States; University of Massachusetts, Boston, Boston, Massachusetts, United States; Mississippi State Department of Health, Ridgeland, Mississippi, United States

## Abstract

Measures of population health can be a tool to advance healthy equity, because they identify disparities that may otherwise go undetected. In this study, we examined if community rates of sensory impairments (vision and/or hearing difficulty) had any association with self-reported cognitive difficulties among older adults. Understanding the distribution of multiple conditions that impact function and independent living may identify geographic hotspots in need of supportive interventions. We describe the prevalence of visual difficulty and hearing difficulty among Mississippi adults age 65 years and older and examine the association of visual difficulty and hearing difficulty with cognitive difficulty in older adults at the community level in 82 counties in Mississippi. Small area estimation techniques were used to calculate age and gender adjusted community rates using three data sources: the American Community Survey (2016-2020), the Behavioral Risk Factor Surveillance System (2017-2020), and CMS Medicare Beneficiary Summary Files (2018). Results show there are 8.7% of older adults 65+ with self-reported vision difficulty and 15.5% with self-reported hearing difficulty. The multiple linear regression models after adjusting for age, gender, race, marital status, education, income, and chronic diseases, counties with higher rates of vision difficulty and hearing difficulty were significantly associated with higher rates of cognitive difficulty compared to those with lower prevalence (vision difficulty: β=0.36, p< 0.001, hearing difficulty: β=0.32, p< 0.001). Implications suggest the need for practitioners and policymakers to establish cognitive difficulty-related interventions targeted to service areas with a high prevalence of sensory difficulties.

